# Not All Fevers in the Neuro ICU Are Neurogenic: An Uncommon Cause That Should Be Considered — Buried Bumper Syndrome

**DOI:** 10.51894/001c.164416

**Published:** 2026-08-01

**Authors:** Kesava Manikanta Achuta, Sai Tharun Reddy Gopannagari, Monika Kadari, Narissa Nursjamsi, Ashutosh Suresh, Abhishek Vadher, Sujata Kambhatla, Pranay Gupta

**Affiliations:** 1 Garden City Hospital

## 55

### CASE DESCRIPTION

A 43-year-old female was admitted after a motor vehicle collision with subarachnoid hemorrhage. Bilateral posterior communicating artery aneurysms were treated with endovascular coiling, and delayed cerebral ischemia due to severe vasospasm required intra-arterial verapamil therapy. She underwent tracheostomy and PEG tube placement for long-term nutrition. Within a week, persistent fevers developed. Infectious evaluation failed to identify a source, and Doppler ultrasound revealed extensive bilateral lower extremity and right upper extremity deep venous thrombosis. Tachycardia, tachypnea, and CT pulmonary angiography showing submassive pulmonary embolism prompted anticoagulation with intravenous heparin. Despite dose escalation, therapeutic levels were not achieved, raising concern for heparin resistance, and the patient was transitioned to argatroban infusion. Persistent fevers led to a repeat physical examination revealing fluctuant swelling of the left upper abdominal wall. CT imaging showed the PEG tube bulb was positioned outside the gastric lumen in the anterior abdominal wall, with oral contrast extravasation and extensive soft-tissue emphysema. General surgery consulted, and the patient underwent incision and drainage of an abdominal wall collection. Surgical exploration revealed purulent material mixed with enteral feeding contents, confirming an abdominal wall abscess associated with buried bumper syndrome. The PEG tube was removed, and the patient received antibiotics, parenteral nutrition, and local wound care. Follow-up imaging showed infection resolution and gradual spontaneous closure of the gastrocutaneous fistula.

### DISCUSSION

Buried bumper syndrome (BBS), an uncommon complication of PEG tube placement (0.3–2.4%), occurs when bumper tension compresses the gastric wall, causing mucosal ischemia and internal migration into the gastrostomy tract or abdominal wall. Imaging shows bumper displacement outside the gastric lumen, with complications like cellulitis & abscesses. Management depends on migration extent: endoscopic if bumper within the stomach, surgical removal and drainage if bumper beyond the stomach or abscesses. Mechanisms for Acquired Heparin Resistance include elevated acute-phase reactants, reduced antithrombin activity, and increased heparin-binding proteins. Direct thrombin inhibitors (e.g., argatroban) for heparin resistance (4–26% of critically ill patients) is needed if no response to heparin. Careful PEG tube positioning, maintaining a 1 cm space between the external bumper and skin, and regular site inspection reduce BBS and complications.

